# Transcriptional differences between coronavirus disease 2019 and bacterial sepsis

**DOI:** 10.1186/s12985-022-01930-y

**Published:** 2022-11-28

**Authors:** Hiroshi Ito, Masakazu Ishikawa, Hisatake Matsumoto, Fuminori Sugihara, Daisuke Okuzaki, Haruhiko Hirata, Hiroshi Ogura

**Affiliations:** 1 Department of Traumatology and Acute Critical Medicine, Osaka University Graduate School of Medicine, Osaka University, 2-15 Yamada-Oka, Suita-Shi, Suita-City, Osaka, 565-0871 Japan; 2 Laboratory for Human Immunology (Single Cell Genomics), WPI Immunology Frontier Research Center, Osaka University, Suita-City, Osaka, Japan; 3 Genome Information Research Center, Research Institute for Microbial Diseases, Osaka University, Suita-City, Osaka, Japan; 4 Institute for Open and Transdisciplinary Research Initiatives, Osaka University, Osaka, Japan; 5 Center for Infectious Disease Education and Research (CiDER), Osaka University, Osaka, Japan; 6 Core Instrumentation Facility, Immunology Frontier Research Center and Research Institute for Microbial Disease, Osaka University, Suita-City, Osaka, Japan; 7 Department of Respiratory Medicine and Clinical Immunology, Osaka University Graduate School of Medicine, Suita-City, Osaka, Japan

**Keywords:** Coronavirus disease 2019, Severe acute respiratory syndrome coronavirus 2, Neutrophil, Mitochondria

## Abstract

**Background:**

Coronavirus disease 2019 (COVID-19), caused by severe acute respiratory syndrome coronavirus 2, has led to major public health crises worldwide**.** Several studies have reported the comprehensive mRNA expression analysis of immune-related genes in patients with COVID-19, using blood samples, to understand its pathogenesis; however, the characteristics of RNA expression in COVID-19 and bacterial sepsis have not been compared. The current study aimed to address this gap.

**Methods:**

RNA-sequencing and bioinformatics analyses were used to compare the transcriptome expression of whole blood samples from patients with COVID-19 and patients with sepsis who were admitted to the intensive care unit of Osaka University Graduate School of Medicine.

**Results:**

The COVID-19 and sepsis cohorts showed upregulation of mitochondrial- and neutrophil-related transcripts, respectively. Compared with that in the control cohort, neutrophil-related transcripts were upregulated in both the COVID-19 and sepsis cohorts. In contrast, mitochondrial-related transcripts were upregulated in the COVID-19 cohort and downregulated in the sepsis cohort, compared to those in the control cohort. Moreover, transcript levels of the pro-apoptotic genes *BAK1*, *CYCS*, *BBC3*, *CASP7*, and *CASP8* were upregulated in the COVID-19 cohort, whereas those of anti-apoptotic genes, such as *BCL2L11* and *BCL2L1*, were upregulated in the sepsis cohort.

**Conclusions:**

This study clarified the differential expression of transcripts related to neutrophils and mitochondria in sepsis and COVID-19 conditions. Mitochondrial-related transcripts were downregulated in sepsis than in COVID-19 conditions, and our results indicated suboptimal intrinsic apoptotic features in sepsis samples compared with that in COVID-19 samples. This study is expected to contribute to the development of specific treatments for COVID-19.

**Supplementary Information:**

The online version contains supplementary material available at 10.1186/s12985-022-01930-y.

## Background

Coronavirus disease 2019 (COVID-19), caused by severe acute respiratory syndrome coronavirus 2 (SARS-CoV-2) [1], was first reported in China in December 2019 [2] and rapidly spread worldwide. COVID-19 was declared as a pandemic by the World Health Organization (WHO) in March 2020. As of February 28, 2022, more than 437 million people had been infected, and more than 5.9 million died from the disease [3]. COVID-19 is characterized by respiratory symptoms, with approximately 15% of patients developing pneumonia and 5% developing respiratory failure due to acute respiratory distress syndrome, shock, or multiple organ failure [4]. The respiratory disturbances observed in many patients with COVID-19 admitted to the intensive care unit are reported to have an aggressive immune response [5, 6]; however, the currently available data on this effect remain incomplete. Thus, studies aimed at clarifying the pathogenesis of COVID-19 are urgently needed.

Viruses and bacteria that enter the bloodstream bind to pattern recognition receptors, such as Toll-like receptors (TLRs), on immune cells as pathogen-associated molecular patterns [6]. Intracellular transcription factors activated by pattern recognition receptor stimulation bind to nuclear DNA and transcribe mRNA. Ultimately, the translated cytokines and other proteins are released into the bloodstream, leading to systemic inflammation. Excessive inflammation leads to severe conditions, ranging from systemic inflammatory response syndrome to multiple organ failure [7–10]. COVID-19 induces inflammation mainly through TLR3 and TLR7/8. In contrast, pathogenic bacteria stimulate all TLRs except for TLR3 [11]. Differences in host immune responses between COVID-19 and pathogenic bacteria have thus been reported. Although some studies have reported the comprehensive mRNA expression of COVID-19 using blood samples to unravel the molecular mechanisms [12, 13], the characteristics of COVID-19 RNA expression compared with those of sepsis caused by bacterial infection have not been widely examined. This study aimed to compare the whole blood transcriptomes of patients with COVID-19 (caused by SARS-CoV-2) and patients with sepsis (caused by bacteria) who were admitted to the intensive care unit of Osaka University Graduate School of Medicine between July 2020 and February 2021, to identify the transcripts that are differentially expressed under COVID-19 and sepsis conditions. This study will improve our understanding of the molecular mechanisms of COVID-19 and bacterial sepsis and provide insights into the differential host responses to these conditions.

## Methods

### Study design and participants

We performed a prospective, observational, single-center study at the Osaka University Graduate School of Medicine (Osaka, Japan). The study protocol complied with the principles of the Declaration of Helsinki and was approved by the Institutional Review Board of Osaka University Hospital (Permit Number: 885 [Osaka University Critical Care Consortium Novel Omix Project; Occonomix Project]). Written informed consent was obtained from the patients or their relatives and healthy volunteers to collect blood samples.

The first cohort (N = 40) comprised patients with COVID-19 who were admitted to the intensive care unit of Osaka University Graduate School of Medicine between July 2020 and February 2021. All patients were diagnosed with COVID-19 using SARS-CoV-2 RT-PCR testing and pneumonia using chest computed tomography. Patients were classified by ordinal score 0–8 based on the WHO ordinal scale [14]: 0, no clinical or virological evidence of infection; 1, no limitation of activities; 2, limitation of activities; 3, hospitalized, no oxygen therapy; 4, oxygen by mask or nasal prongs; 5, non-invasive ventilation or high-flow oxygen; 6, intubation and mechanical ventilation; 7, ventilation + additional organ support-pressors, renal replacement therapy (RRT), extracorporeal membrane oxygenation (ECMO); 8, death. All patients in this cohort met the ordinal scale of 4–8.

The second cohort (N = 18) comprised patients diagnosed with sepsis and bacterial infection who were admitted to the Department of Traumatology and Acute Critical Medicine, Osaka University Graduate School of Medicine, between August 2020 and February 2021. All patients in this cohort met the diagnostic criteria for Sepsis-3 [15] and were diagnosed with bacterial infections based on culture tests. The control population consisted of outpatients who were enrolled via public poster advertisements.

The third cohort (N = 16) comprised the individuals who visited the hospital in healthy condition.

### Sample collection and clinical data

Samples from cohorts 1 and 2 were collected on the first or second day (within 24 h) of admission of the patients to our hospital, and those from the third cohort were collected on days with no physical problems. The collection tubes containing the blood samples were stored at − 30 °C until analysis.

The clinical data collected from the electronic medical records of the patients by the investigators included age, sex, body mass index, Acute Physiology and Chronic Health Evaluation II (APACHE II) score, Sequential Organ Failure Assessment (SOFA) score, comorbid conditions (hypertension, diabetes, and hyperlipidemia), and hospital outcomes.

### Statistical analyses of clinical data

Summary data are presented as medians (interquartile range) for continuous variables and numbers (%) for categorical variables. The chi-square test and Fisher’s exact test were used for binary variables to compare the comorbidity between COVID-19 cohort and sepsis cohort. Statistical analyses were performed using commercially available statistical analysis software (JMP Pro 16 software, SAS Institute Inc., Cary, NC, USA). Statistical significance was set at *P* < 0.05.

### Whole blood RNA isolation and library construction

Total RNA was isolated from whole blood using the PAXgene™ Blood RNA System (BD Biosciences, Franklin Lakes, NJ, USA). The eluted RNA was dissolved in RNase-free water. The quality and quantity of RNA were evaluated using a Bioanalyzer 2100 system (Agilent Technologies, Santa Clara, CA, USA). Double-stranded cDNAs were synthesized from the RNA, and libraries were prepared using the SMART-seq HT kit (Takara, Shiga, Japan) according to the manufacturer's protocol. The libraries were quantified using the Illumina Library Quantification Kit (Kapa Biosystems, Wilmington, MA, USA), and the fragment size distribution was determined using the Bioanalyzer 2100 system (Agilent Technologies, Santa Clara, CA, USA).

### RNA-sequencing and bioinformatics analysis

High-throughput sequencing was performed using an MGIseq 2000 system (MGI Tech Co., Ltd., Shenzhen, China) with 100-bp paired-end reads, which were converted into Fastq files. Tophat2 v2.1.1 [16] was used to read alignments using the human reference genome (hg19). BAM files were converted to raw count files using featureCount v2.0.3 [17]. The raw counts were analyzed using iDEGES/edgeR in the TCC package (version 1.36.0) [18]. Differentially expressed transcriptomes were evaluated for COVID-19 vs. sepsis, healthy control vs. COVID-19, and healthy control vs. sepsis, using a false discovery rate cut-off of 0.1. Gene Ontology (GO) and KEGG enrichment analysis was conducted using the R-package clusterProfiler v.4.4.4 [19].

## Results

### Patient characteristics

Table 1 shows an overview of the patient characteristics. The median ages of patients in the COVID-19, sepsis, and control cohorts were 72, 81, and 47 years, respectively. The body mass index values were 23.3, 24.2, and 22 kg/m^2^, respectively. The comorbidity of diabetes was significantly higher in the COVID-19 cohort than in the sepsis cohort (*P* = 0.0047). All patients with COVID-19 were treated in intensive care units, and the mortality rate of these patients was 12.5%.

**Table 1:** Clinical characteristics of the participants in the three cohorts

	COVID-19 (N = 40)	Sepsis (N = 18)	Healthy control (N = 16)
Age (IQR) years	72 (70.75–76.00)	81 (73.25–3.75)	47 (34.00–55.50)
Male sex: number (%)	27 (67.5)	14 (77.8)	8 (50.0)
BMI (IQR) kg/m^2^	23.7 (22.5–25.9)	24.2 (20.4–27.5)	23.1 (20.7–26.7)
APACHE II score (IQR)	13 (10–18)	21 (14–26)	–
SOFA score (IQR)	5 (3–7)	8 (4–12)	–
Comorbidity: number (%)			
Hypertension	21 (52.5)	7 (38.9)	2 (12.5)
Hyperlipidemia	6 (15.0)	3 (16.7)	6 (37.5)
Diabetes	20 (50.0)	2 (11.1)	1 (6.3)
Hospital death: number (%)	5 (12.5)	4 (22.2)	-

*APACHE II* acute physiology and chronic health evaluation II, *BMI* body mass index, *COVID-19* coronavirus disease 2019, *IQR* interquartile range, *SOFA* sequential organ failure assessment

### Differential transcriptome expression analysis between patients with COVID-19 and sepsis

**Fig. 1:**
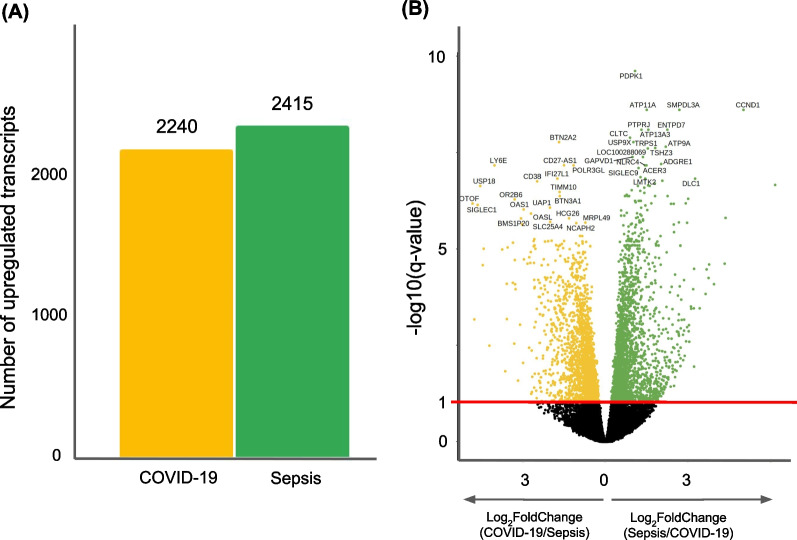
Differentially expressed transcriptomes between patients with coronavirus disease 2019 (COVID-19) and sepsis. **A** Number of differentially expressed transcripts between the COVID-19 and sepsis. **B** Volcano plot describing differentially expressed transcripts between the two disease groups. The transcripts at a false discovery rate cut-off of 0.1 are shown in orange or green. The top 20 transcripts with the lowest false discovery rate are labeled

**Fig. 2:**
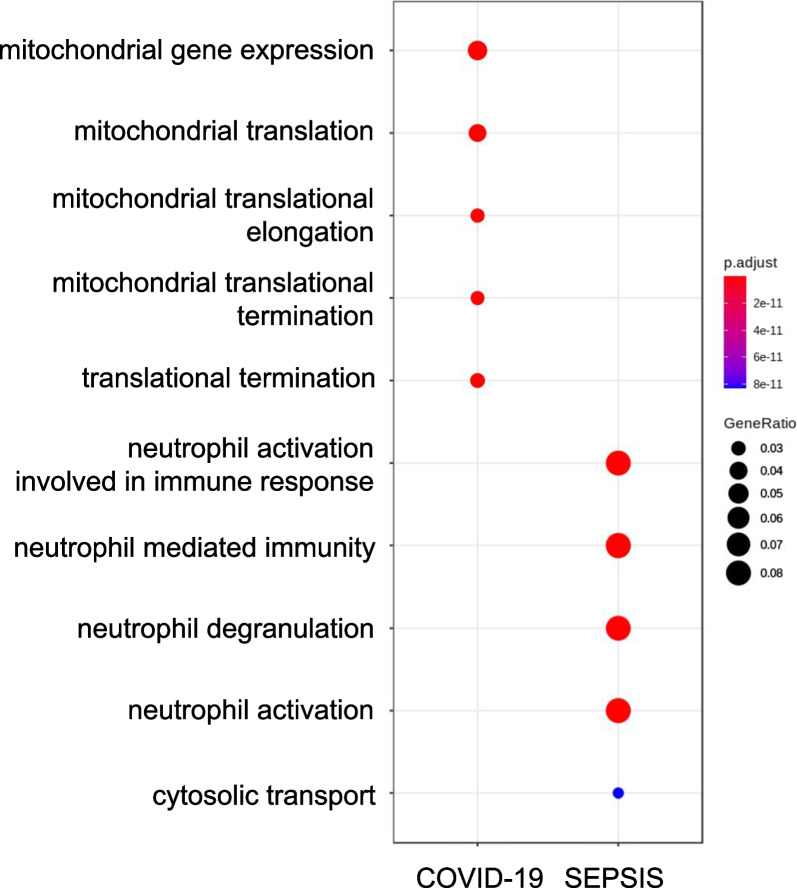
Dot plot of most enriched GO terms (top five) significantly upregulated in COVID-19 or sepsis. The size of the dots represents the gene ratio, and the color of the dots represents the adjusted *P*-value. Abbreviations: COVID-19, coronavirus disease 2019; GO, Gene Ontology

**Fig. 3:**
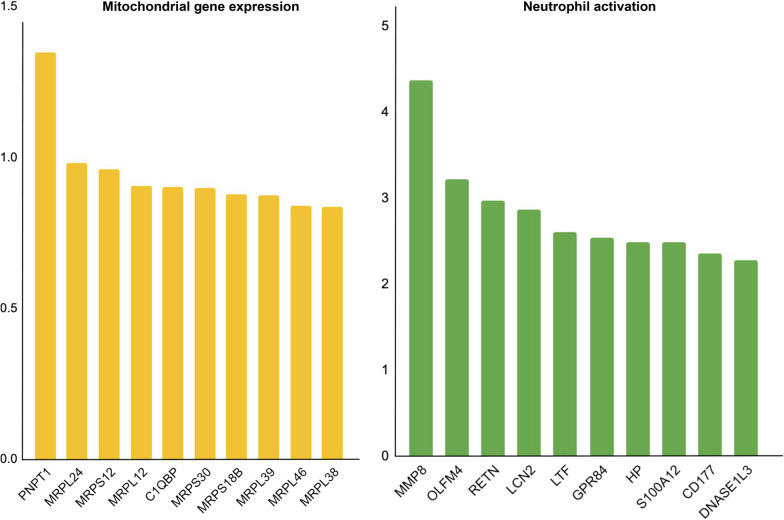
Top differentially expressed transcripts in the “mitochondrial gene expression” and “neutrophil activation” terms. The transcripts related to the “mitochondrial gene expression” term that are upregulated in the COVID-19 group and those related to the “neutrophil activation” term that are upregulated in the sepsis group are shown. Abbreviation: COVID-19, coronavirus disease 2019

**Fig. 4:**
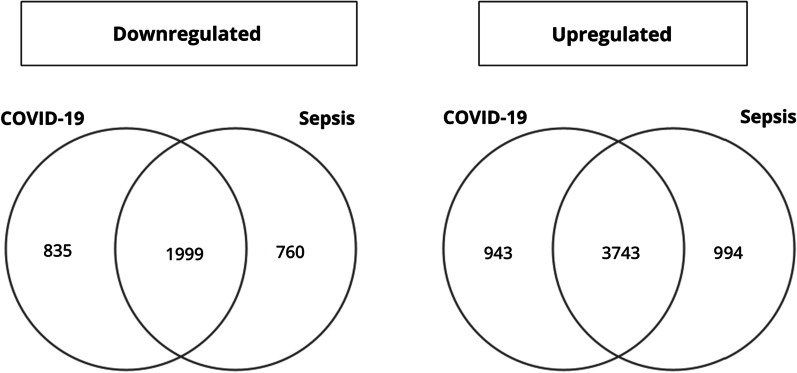
Venn diagram of differentially expressed transcriptomes between the disease and healthy control groups. The overlapping region shows the number of transcripts differentially expressed in both diseases compared to those in healthy controls. Abbreviation: COVID-19, coronavirus disease 2019

**Fig. 5:**
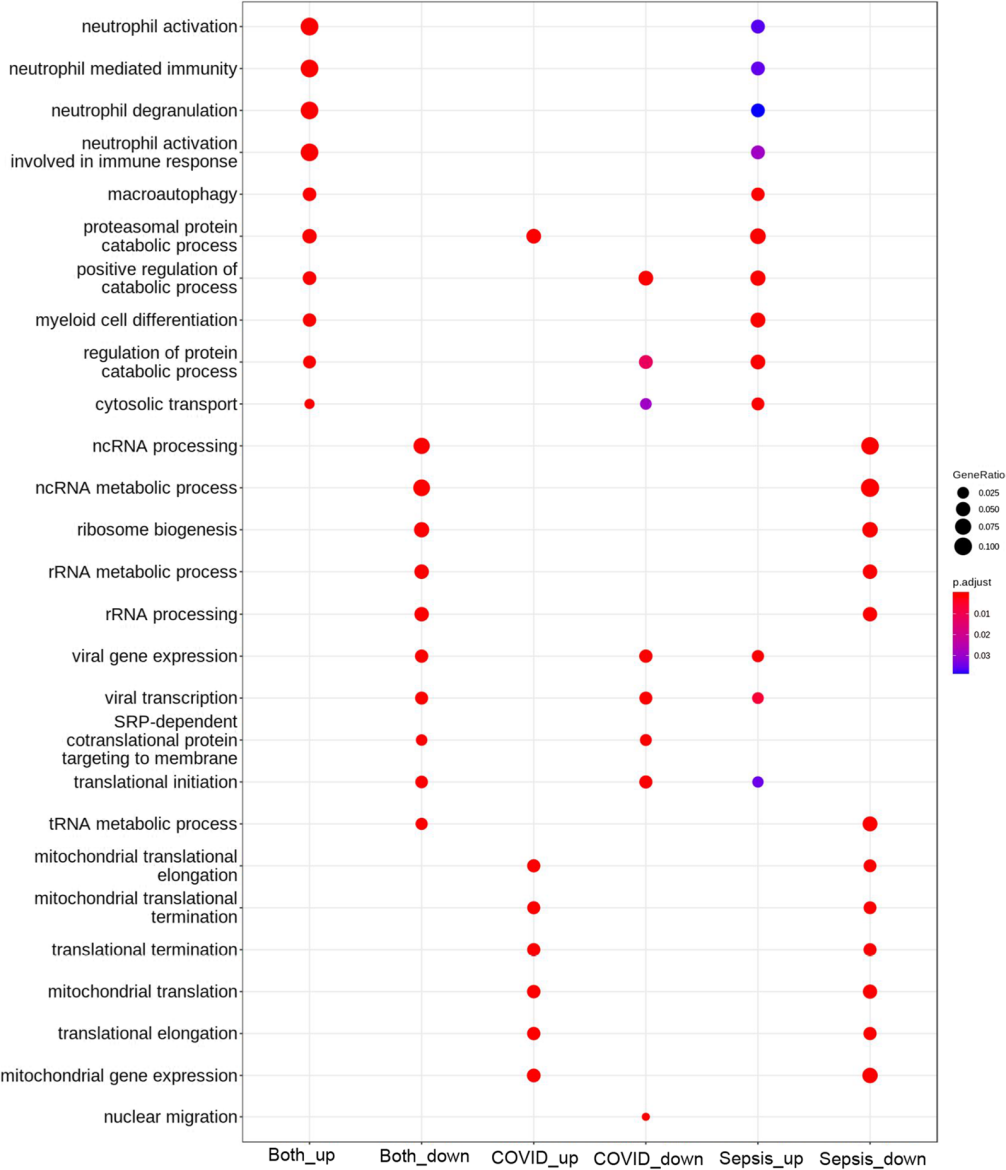
Dot plot of enriched terms for differentially expressed transcripts between the disease and control groups. The top five most enriched GO terms are shown. The size of the dots represents the gene ratio, and the color of the dots represents the adjusted *P*-value. “Both_up” and “Both_down” means the transcript groups that are up- or downregulated, respectively, in both COVID-19 and sepsis groups compared with that in the healthy control group. “COVID_up” and “COVID_down” represent the transcript groups that are up- or downregulated, respectively, specifically in the COVID-19 group. “sepsis_up” and “sepsis_down” represent the transcripts that are up- or downregulated, respectively, specifically in the sepsis group. Abbreviations: COVID-19, coronavirus disease 2019; GO, Gene Ontology

We conducted differential expression analysis of whole blood transcriptomes between COVID-19 and sepsis patients admitted to the same hospital; the results are shown in Fig. 1 and Additional file 1. GO enrichment analysis of the differentially expressed transcripts revealed that terms related to “mitochondria” were highly enriched in the upregulated transcripts in COVID-19 samples, whereas terms related to “neutrophil” were highly enriched in sepsis samples (Fig. 2). Among the transcripts related to the term “mitochondrial gene expression,” polyribonucleotide nucleotidyltransferase 1*(PNPT1)* showed the highest upregulation in the COVID-19 cohort, followed by *MRPL24* and *MRPS12* (Fig. 3). Concerning the transcripts related to “neutrophil activation,” matrix metalloproteinase 8 (*MMP8*) showed the highest upregulation in sepsis samples, followed by olfactomedin 4 (*OLFM4*) and resistin (*RETN*) (Fig. 3). We also compared the transcriptome expression profiles of the patients in each disease cohort with that of the healthy control cohort. The differential expression analysis identified a total of 1999 and 3743 transcripts that were down- and upregulated in both the COVID-19 and sepsis cohorts, respectively. Additionally, 835 and 760 transcripts were down- and upregulated, respectively, only in the COVID-19 cohort, while 943 and 994 transcripts were down- and upregulated, respectively, only in the sepsis cohort (Fig. 4, Additional file 3). The results of the GO enrichment analysis of these transcripts are shown in Fig. 5 and Additional file 4. For the transcripts upregulated in both diseases, the term “neutrophil activation” (GO:0042119) was most enriched (q-value = 1.65 × 10^−115^), followed by “neutrophil-mediated immunity” (GO:0002446, q-value = 3.39 × 10^−115^) and “neutrophil degranulation” GO:0043312, q-value = 1.80 × 10^−114^). For transcripts downregulated in both diseases, the term “ncRNA processing” (GO:0034470) was the most enriched term (q-value = 7.88 × 10^−34^). For transcripts upregulated only in COVID-19, the term “mitochondrial translational elongation” (GO:0070125) showed the greatest enrichment (q-value = 1.56 × 10^−14^), followed by “mitochondrial translational termination” (GO:0070126, q-value = 1.22 × 10^−13^), “translational termination” (GO:0006415, q-value = 9.06 × 10^−13^), “mitochondrial translation” (GO:0032543, q-value = 1.09 × 10^−11^), “translational elongation” (GO:0006414, q-value = 6.82 × 10^−11^), and “mitochondrial transcriptome expression” (GO:0140053, q-value = 6.82 × 10^−11^). These terms were also enriched in the transcripts that were downregulated only in sepsis. Our results depict that neutrophil-related transcripts were upregulated in both diseases. In contrast, mitochondrial-related transcripts were downregulated in sepsis samples and upregulated in COVID-19 samples in comparison with the COVID-19 vs. sepsis groups as well as the disease vs. control groups.

### Transcripts related to intrinsic apoptosis

**Fig. 6:**
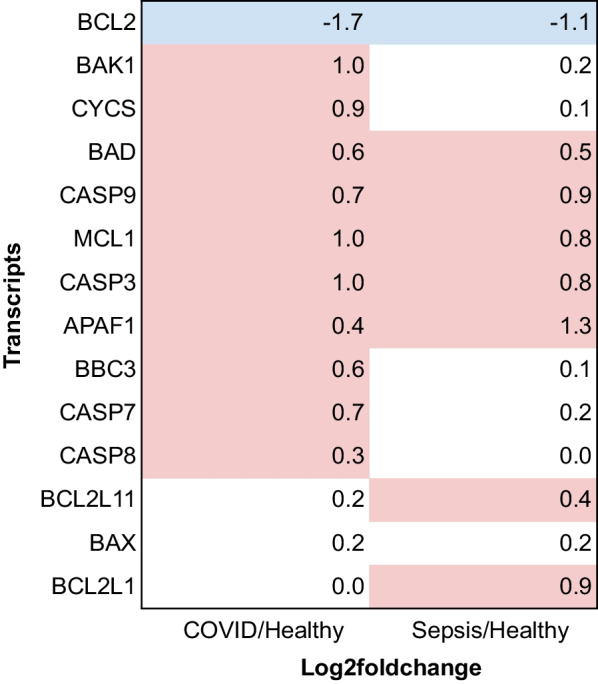
Log_2_ fold-change of transcripts related to intrinsic apoptosis. Transcripts with q-values of < 0.1 are shown in red or blue. Abbreviations: COVID-19, coronavirus disease 2019

Mitochondria play key roles in intrinsic apoptosis, which is triggered by cytochrome c released from the mitochondria by mitochondrial outer membrane permeabilization [20]. We next focused on transcripts involved in apoptotic processes and identified that the GO terms related to mitochondrial outer membrane permeabilization were enriched only in the COVID-19 group (GO:1902686, GO:0097345, GO:1901028; Additional file 4). As shown in the heatmap in Fig. 6, the transcript levels of several pro-apoptotic genes, namely *BAK1*, *CYCS*, *BBC3*, *CASP7*, and *CASP8*, were upregulated (shown in red) in the COVID-19 group but not in the sepsis group. In contrast, the transcript levels of anti-apoptotic genes, such as *BCL2L11* and *BCL2L1*, were upregulated only in the sepsis group, wherein that of *BCL2* was downregulated in both the groups. These results suggest that intrinsic apoptosis occurs in patients with COVID-19 but is incomplete in those with sepsis. Berthenet et al. showed that cells with suboptimal apoptosis, referred to as failed apoptosis, exhibit specific transcriptional signatures [21]; therefore, the GO terms described by these authors were investigated in the present study. Among the five GO terms related to failed apoptosis, four were enriched in transcripts upregulated in sepsis but not in COVID-19 samples (Table 2). These results indicate that sepsis samples tend to have apoptotic features.

**Table 2:** KEGG terms that are signatures of failed apoptosis, as determined by Berthenet et al. [21]

KEGG ID	KEGG term	COVID-19		Sepsis	
		Genes	q-value	Genes	q-value
hsa04510	Focal adhesion	Not enriched	–	Upregulated	0.015085
hsa04514	Cell adhesion molecules (CAMs)	Not enriched	–	Not enriched	-
hsa04010	MAPK signaling pathway	Not enriched	–	Upregulated	0.001412
hsa04670	Leukocyte transendothelial migration	Not enriched	–	Upregulated	0.000036
hsa04810	Regulation of actin cytoskeleton	Not enriched	–	Upregulated	0.000009

*COVID-19* coronavirus disease 2019, *KEGG* Kyoto Encyclopedia of Genes and Genomes, *MAPK* mitogen-activated protein kinase

## Discussion

We compared the differential expression of the transcriptome between patients with COVID-19 and those with bacterial sepsis to identify the characteristics of transcripts expressed under specific disease conditions. The findings demonstrated that transcripts related to mitochondria were upregulated in COVID-19 samples, whereas those related to neutrophils were upregulated in sepsis samples. However, compared with that in healthy controls, the transcripts related to neutrophils were upregulated in both diseases, but mitochondrial-related transcripts were upregulated in COVID-19 and downregulated in sepsis. These results show that the expression of mitochondrial-related transcripts significantly differed between the two diseases.

Neutrophils are the most abundant cell type in circulating leukocytes and among the first cells recruited to the infection site. These cells are key in shaping the early response to a pathogen and in mediating the innate and adaptive arms of the immune system [22]. A previous study based on RNA-sequencing analysis has reported that neutrophil-related transcripts are upregulated in both patients with COVID-19 and those with sepsis [13]; however, reports on the differences in neutrophil-related transcript expression between these diseases are scarce.

Here, we showed that the expression of neutrophil-related transcripts was the highest in patients with sepsis, followed by that in patients with COVID-19 and healthy controls. The most upregulated gene in the “neutrophil activation” term in sepsis was *MMP8*, which encodes a member of the MMP family of proteolytic enzymes that play multiple roles in the immune response to infection [23]. Knockout of *MMP8* reduces bacterial clearance, and MMP is activated not only by host cells but also by bacterial proteases [24]. Additionally, *MMP8* expression is upregulated in sepsis and COVID-19 samples [25]. However, our result revealed increased expression of *MMP8* in sepsis than in COVID-19 samples, indicating that *MMP8* expression was highly upregulated by bacteria in sepsis samples. The second and third most upregulated transcripts were *LCN2* and *LTF*, respectively, which encode iron-binding proteins. LCN2 mediates the innate immune response to bacterial infection by sequestering iron [24]. Taken together, it can be inferred that innate immunity corresponding to neutrophils may be adapted by bacterial rather than viral infections.

Transcripts showing the largest differences between COVID-19 and sepsis samples were related to mitochondria, particularly the inner membrane and matrix. Expression of the mitochondrial-related transcripts in COVID-19 and sepsis showed opposite directions of expression compared to that in normal subjects. RNA viruses, such as SARS-CoV-2, are recognized by retinoic acid-inducible gene (RIG)-like receptors [26]. A study using *Atg5*^*−/−*^ cells revealed that RIG-like receptors are enhanced by mitochondrial reactive oxygen species [27, 28]. As our results show that all RIG-like receptor transcriptomes were upregulated in COVID-19 (Additional file 3), we suggest that antiviral immunity may occur via the activation of mitochondrial activity. RIG-like receptors typically activate mitochondrial antiviral-signaling proteins, which induces cytokine secretion [26]. However, several reports showed that membrane proteins or the nucleocapsid protein of SARS-CoV-2 inhibit mitochondrial antiviral-signaling proteins, and therefore, the virus can antagonize viral immunity [26, 29]. Our results also revealed the downregulation of mitochondrial antiviral-signaling proteins (Additional file 3). These results indicate that although host cells enhance viral immunity by activating mitochondrial activity, SARS-CoV-2 can infect a host cell by inhibiting mitochondrial antiviral-signaling protein activity.

Studies have suggested that bacterial sepsis-related organ failure is related to mitochondrial dysfunction and a lack of bioenergetic recovery [30, 31], with some reports suggesting a decrease in the cellular energy supply by mitochondria [30]. Concordantly, we observed a decreased expression of mitochondrial-related genes in sepsis. Moreover, we found that the apoptotic process was suboptimal in sepsis samples compared to that in COVID-19 samples. In bacterial sepsis, BCL2-associated X and BCL2 antagonist/killer 1, which function as outer membrane components of the mitochondrial permeability pore [32], did not show differential expression (Fig. 5). Additionally, cytochrome c expression was not upregulated in the sepsis group compared with that in the COVID-19 and healthy control groups (Fig. 5). These results indicate that the amount of cytochrome c is insufficient during suboptimal apoptosis in sepsis. We also found that the expression of *PNPT1,* which encodes a key enzyme in mitochondrial RNA metabolism, was downregulated in the sepsis group—the loss of the activity of PNPT1 results in combined respiratory chain deficiency [33]. Moreover, *PNPT1* knockdown inhibits apoptotic RNA decay and reduces apoptosis [34]. Therefore, *PNPT1* downregulation in sepsis supports the failed apoptosis in sepsis samples.

This study has several limitations. First, this was a single-center study, and the number of participants was small. The higher comorbidity of COVID-19 and diabetes mellitus in patients with COVID-19 compared with that in patients with sepsis indicated that diabetes may have influenced the differences in neutrophil and mitochondrial transcripts in the two disease groups [35, 36].

## Conclusions

We performed whole blood transcriptome analysis to investigate the differences in transcriptome expression in patients with COVID-19 and bacterial sepsis. Our results suggest that neutrophils and mitochondria influence the differential expression of the transcriptome in COVID-19 and bacterial sepsis conditions. These findings provide insights into the differences in the differential immune responses of the host to the source of infection at the molecular level and may contribute to developing a specific treatment for COVID-19.

## Supplementary Information

**Additional file 1**. Table S1.

**Additional file 2**. Table S2.

**Additional file 3**. Table S3.

**Additional file 4**. Table S4.

## Data Availability

The raw data have been deposited to Gene Expression Omnibus under the accession numbers GSE199816 and GSE179850 for future access.

## Abbreviations

COVID-19: Coronavirus disease 2019
SARS-CoV-2: Severe acute respiratory syndrome coronavirus 2
WHO: World Health Organization
TLR: Toll-like receptor
APACHE II: Acute Physiology and Chronic Health Evaluation II
SOFA: Sequential Organ Failure Assessment
GO: Gene Ontology
PNPT1: Polyribonucleotide nucleotidyltransferase 1
MMP: Matrix metalloproteinase
LCN2: Lipocalin 2
LTF: Lactotransferrin
RIG: Retinoic acid-inducible gene
RRT: Renal replacement therapy
ECMO: Extracorporeal membrane oxygenation
